# 40 Inflamm-aging, Burns and the Gut-lung Axis in Mice

**DOI:** 10.1093/jbcr/irae036.040

**Published:** 2024-04-17

**Authors:** Elizabeth J Kovacs, Devin M Boe, Daniel Frank, Juan-Pablo Idrovo, Rachel H McMahan, Travis M Walrath, Kevin M Najarro

**Affiliations:** University of Colorado Denver, Aurora, Colorado; University of Colorado Denver, Aurora, Colorado; University of Colorado Denver, Aurora, Colorado; University of Colorado Denver, Aurora, Colorado; University of Colorado Denver, Aurora, Colorado; University of Colorado Denver, Aurora, Colorado; University of Colorado Denver, Aurora, Colorado

## Abstract

**Introduction:**

The elderly have a heightened basal state of inflammation, known as “inflamm-aging,” which predisposes them to aberrant immune responses and may reduce their ability to withstand burn trauma. We and others believe that a combination of factors released from the burn site in the aged host trigger the gut to become more permeable and, along with shifts in fecal microbiota, sets the stage for excessive systemic and pulmonary inflammatory responses and poor prognosis after injury.

**Methods:**

We used our well-established, clinically relevant murine model of burn injury in which young and aged mice are subjected to a 12% total body surface area dorsal scald burn or sham injury. At 24 hours after injury, mice were humanely euthanized and lungs, blood, ileum and feces were collected for analysis including histopathology, RNA sequencing, 16S rRNA gene sequencing, and real-time PCR.

**Results:**

There were no differences in distribution of lung myeloid cell populations between groups; all samples were composed of >97% CD45+Ly6g-CD11c+SiglecF+ alveolar macrophages (AM). However, there were profound changes in transcriptional profiles of these cells both before and after injury. Compared with AM from young sham mice, AMs from young, burned mice displayed diverse and complex transcriptional changes, totaling 964 differentially expressed genes (cut-off p < 0.01; FDR < 0.05). In contrast, injury produced fewer changes in mRNA expression of AMs from aged mice relative to that of sham age-matched controls, with changes in only 216 genes. The genes differentially expressed with age included those involved in endosomal/lysosomal trafficking, antigen presentation, tissue remodeling, and receptors or secreted factors related to immune responses, possibly altering their ability to respond appropriately to invading pathogens. Since the gut is a motor of critical illness, we also examined the intestine in the same experimental groups. The fecal microbiota of aged-injured mice had significantly less Shannon alpha-diversity when compared to other groups (p < 0.05). This included a dramatic loss in gut-protective bacterial taxa, including Akkermansia muciniphila, in the microbiota of aged-injured mice relative to all other groups. Moreover, there was an 18-fold increase in gut leakiness, using orally gavaged FITC-dextran, when compared to other groups (p < 0.05).

**Conclusions:**

Taken together, these findings suggest that the post-burn loss of protective commensal bacteria and the breach of the intestinal epithelial barrier in aged mice could contribute to the changes in AM gene expression and alter responses to lung infections.

**Applicability of Research to Practice:**

Future studies will explore whether supplementation of protective bacteria, such as Akkermansia muciniphila, after burn injury could restore intestinal and pulmonary homeostasis and AM function in burn patients of all ages.

